# High Frequency and Low Intensity Transcranial Magnetic Stimulation for Smoking Cessation

**DOI:** 10.1155/2021/9988618

**Published:** 2021-09-18

**Authors:** Guadalupe Ponciano-Rodríguez, Carlos A. Chávez-Castillo, Alma E. Ríos-Ponce, Gabriel Villafuerte

**Affiliations:** ^1^Departamento de Salud Pública, Facultad de Medicina, Universidad Nacional Autónoma de México, Mexico City, Mexico; ^2^Clínica Coyoacán, Mexico City, Mexico; ^3^Plan de Estudios Combinados en Medicina (PECEM), Facultad de Medicina, Universidad Nacional Autónoma de México, Mexico City, Mexico

## Abstract

**Introduction:**

Tobacco consumption is one of the main causes of mortality in the world. Because of its effect on health, smoking cessation should be prioritized as an important health intervention; however, current interventions have shown low success rates as only 31% of the cases can stop smoking. In this paper, an intervention with high frequency and low intensity transcranial magnetic stimulation (HFLI TMS) was applied to determine if this type of neuromodulation could have an effect in decreasing tobacco addiction.

**Methods:**

Retrospective data from ten ambulatory smoker patients that underwent 24 sessions of HFLI TMS over 8 weeks were retrieved and are here presented.

**Results:**

Exhaled CO concentrations were statistically significantly different from baseline at the weeks 3, 5, 6, and 8. After the 24 sessions, all patients stopped smoking; this was confirmed directly by exhaled carbon monoxide and the smoking diary. Three months after intervention, eight out of ten subjects continued without smoking. No severe adverse effects were reported by participants.

**Conclusions:**

Overall, employing HFLI TMS appears to have acceptable result; however, further evidence is needed to determine with more certainty its therapeutic effect and adverse effects for addiction intervention.

## 1. Introduction

Tobacco use disorder is one of the main public health problems around the world: about 8 million people die each year as a result of both active and passive exposure to tobacco smoke [1]. Because of these negative effects in health, it is essential to develop effective health interventions that can reduce tobacco consumption and help achieve cessation of consumption. Tobacco cessation, also known as achieving abstinence or quitting, is the goal of pharmacological or behavioral treatments. Abstinence can be further classified by the period of time in which this state prevails, including point prevalence cessation—cessation at a particular point in time—or continuous cessation—avoidance of all tobacco use since quitting day until assessment [2]. However, tobacco cessation is not a simple procedure as only 4% of the people who try to stop smoking can do it by themselves [1]. Currently, first-line therapeutic interventions to help with tobacco cessation include pharmacologic approaches (nicotinic and nonnicotinic medications) and behavioral counseling, mainly cognitive therapy; however, these interventions are only effective in 31% of the cases [3]. Thus, novel interventions targeting different action mechanisms of addictions are in need to increase success rates.

Several noninvasive brain stimulation technologies have been used to treat tobacco addiction with promising results [4, 5]. Among these technologies, repetitive transcranial magnetic stimulation (rTMS), an FDA-approved therapy for treatment-resistant depression, has shown positive effects for smoking cessation as a therapeutic alternative to patients with poor responsiveness to first-line interventions [5]. Epidemiological, clinical, and animal research suggests that depression and addiction share a large degree of overlap among their neurobiological substrates [6]. Originally designed for depression, interventions, including rTMS, have been used successfully to treat addiction [5], and interventions directed to treat addiction also have antidepressant effects [6, 7]. In fact, after a recent pivotal trial in which rTMS was compared against sham stimulation and showed positive results for smoking cessation, the FDA approved the application of rTMS as an aid in short-term smoking cessation for adults [8].

Classical rTMS devices apply high intensity magnetic pulses (near 1 tesla) over the skull at frequencies ranging from 1 to 50 Hz. As magnetic permeability of biological tissues is similar to that of air, the rapidly changing magnetic field travels across the bone and induces an electric current in the brain's cortex.

The current paradigm in rTMS states that a high magnitude magnetic field is required in order to generate biological cortical changes [9]; however, several studies have proved that magnetic field intensities several orders of magnitude lower than current rTMS protocols, but at higher frequencies, produce measurable changes in cortical excitability [10], brain glucose metabolism [11], and cognitive functioning [12].

It is of great interest to apply high frequency and low intensity (HFLI) TMS as these devices offer unique characteristics, such as portability and safety, as well as a more accessible price; these characteristics could potentially increase patient adherence to treatment and, as a consequence, improve outcome of patients. Thus, we present a case series of 10 patients that sought treatment for smoking cessation and HFLI TMS was used as the only intervention. The objective of this study was to evaluate the smoking cessation rate (point and continuous) after 8 weeks of treatment with HFLI TMS in smokers with moderate-to-severe nicotine dependence; CO exhaled concentration was used as an objective measurement to determine efficacy of intervention and as the main outcome of this study.

## 2. Materials and Methods

### 2.1. Subjects

Health records from a private neuromodulation clinic were screened for ambulatory smoker patients that received at least 24 sessions of HFLI TMS for smoking cessation, without skipping any sessions. Subjects had to smoke at least ten cigarettes per day continuously during one year before the intervention. Patients were not considered for this case series if they had a previous history of substance abuse other than tobacco, chronic diseases out of medical control, morbid obesity, clinically diagnosed mental disorder, and/or epilepsy. Subjects had to have at least 2 months without any active intervention to stop smoking before starting HFLI TMS; subjects that received some type of counseling during the treatment duration were not considered for this study. All clinical data and assessments were retrieved retrospectively from the health records of the clinic. After gathering the records, all patients that completed the previous requirements were contacted and all signed a written informed consent and agreed to the publication of their cases; because of the retrospective nature of the study and data recollection characteristics, no ethics committee approval was required by the institution.

### 2.2. Intervention

The HFLI TMS device described here was designed and manufactured by Actipulse Neuroscience (Boston, USA). In all reported patients, stimulation was applied every third day for 8 weeks with a total of 24 sessions. In each session, HFLI TMS was applied with a circular coil with 60 mm diameter over the left dorsolateral prefrontal cortex. To stimulate this area, the 10–20 EEG coordinates of F3 were measured in the skull of each participant; the center of the coil was located in this spot [13]. Stimulation protocol consisted of 1 continuous train at 575 Hz for 45 minutes with each pulse intensity at around 0.5 milliteslas. During the sessions, patients sat in a comfortable chair and were allowed to move freely without standing up.

### 2.3. Assessments

Clinical history, epidemiological data, Fagerström Test for Nicotine Dependence (FTDN) [13, 14], Test to Assess the Psychological Dependence on Smoking (TAPDS) [15], and Alcohol Use Disorders Identification Test (AUDIT) [16] were acquired or measured before starting the intervention. The Beck Depression Inventory (BDI) [16, 17], Beck Anxiety Inventory (BAI) [18], and the Short Form (SF36) Health Survey [19] were measured before starting the first stimulation session and after the last stimulation session. To evaluate smoking cessation, subjects performed a weekly determination of exhaled carbon monoxide (CO) with a carbon monoxide analyzer using as cutoff points values below 10 ppm to determine smoking cessation [20] (Bedfont Scientific Ltd., Rochester, UK); the same test and criteria were applied to measure continuous abstinence after 3 months of the intervention. Also, patients registered in a diary if they smoked any cigarette during the 8 weeks of stimulation to determine point abstinence and continuous abstinence. The possible adverse effects were assessed after stimulation in each session. Weight and blood pressure were measured weekly. All these assessments, including a copy of the smoking dairy, were retrieved from the clinical record of each patient.

### 2.4. Statistical Analysis

All statistical analyses were performed with IBM SPSS Statistics 20 package for Windows. Figures were created with GraphPad software for Windows. To evaluate changes of exhaled CO concentrations along time, a Friedman test was conducted; afterwards, post hoc pairwise comparisons were performed with a Bonferroni correction for multiple comparisons. Furthermore, related samples' signed-rank tests were used to assess differences between pre- and postintervention of depression symptoms (BDI), anxiety symptoms (BAI), and general health (SF36). Finally, Spearman's rank-order correlation was used to assess relation between changes from baseline in BDI and changes from baseline to week 8 in exhaled CO concentrations, as well as relation between changes from baseline in BAI and changes from baseline to week 8 exhaled CO. Values of *p* < 0.05 were considered statistically significant unless otherwise specified.

## 3. Results and Discussion

### 3.1. Results

Our sample was collected by convenience as only ten different patients matched the previously defined criteria from a total pool of 50 patients that had received HFLI TMS for smoking cessation and other indications in the private clinic. All subjects attended stimulation every third day for 8 weeks receiving a total of 24 sessions. Clinical and epidemiological data of the ten patients are summarized in Table 1. The mean age was 56.80 years (SD ± 9.75); on average, subjects smoked 19.10 cigarettes each day (range 8–40) for 36.90 years (range 20–57) and had attempted 2 times (range 0–5) to quit smoking. We found a moderate-to-very high dependence on nicotine assessed by the FTND score (range 1–9), an AUDIT score of 1.6 (range 0–5), and a moderate-to-high psychological dependence with TAPDS (range 32–63). After the 24 sessions, all patients stopped smoking; this was confirmed directly by exhaled carbon monoxide and the smoking diary, thereby showing achievement of point abstinence. Three months after the intervention was finished, eight out of ten subjects continued without smoking according to personal reports and CO measures, thus showing continuous abstinence. No severe adverse effects were reported by participants. Participants were compliant to each session without reprogramming.

**Figure 1:**
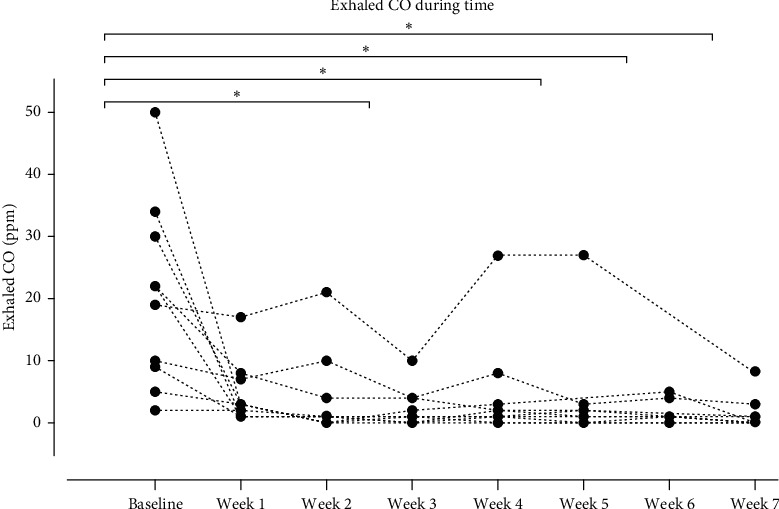
Changes in exhaled CO during the 8-week period of stimulation. There was a statistically significant difference in CO concentration in different time points: from baseline to week 3 (*p* < 0.01), week 5 (*p* < 0.01), week 6 (*p* < 0.01), and last week 7 (*p* < 0.01). CO, carbon monoxide; PPM, parts per million.

To evaluate changes of exhaled CO concentrations along time, a Friedman test was conducted. Exhaled CO concentrations were statistically significantly different at the evaluated time points, *χ*2(7) = 32.409, *p* < 0.01. Post hoc pairwise comparisons were performed with a Bonferroni correction for multiple comparisons; statistical significance was accepted at the *p* < 0.01 level. A statistically significant difference was observed in CO concentration from baseline to week 3 concentration (*p* < 0.01), week 5 concentration (*p* < 0.01), week 6 concentration (*p* < 0.01), and last measured concentration (*p* < 0.01). The results are shown in Figure 1.

**Figure 2:**
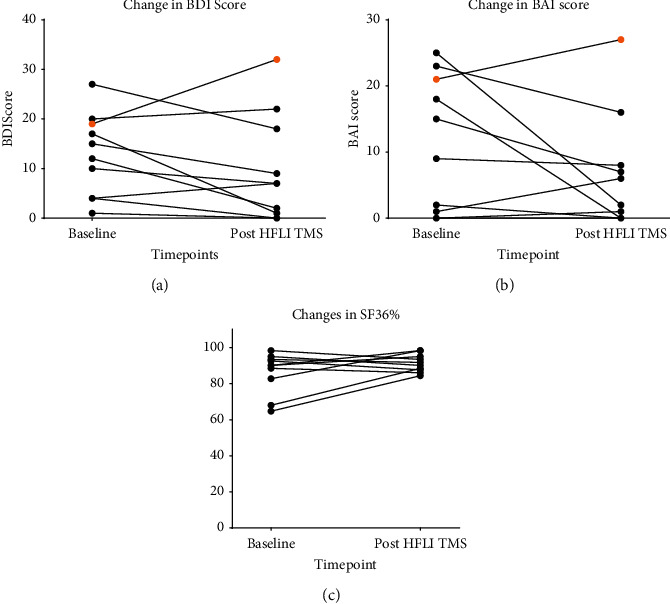
Change in several scores before and after HFLI TMS. Changes in (a) BDI score, (b) BAI score, and (c) SF36% before and after HFLI TMS; no statistical significance was achieved in any of the scores. The orange dot represents subject 7 (see text for more information). BDI, Beck Depression Index; BAI, Beck Anxiety Index; SF36%, percentage of Short Form Health Survey; HFLI TMS, High Frequency and Low Intensity Transcranial Magnetic Stimulation.

Because of small sample size, related samples' signed-rank tests were used to assess differences between pre- and postintervention of depression symptoms (BDI), anxiety symptoms (BAI), and general health (SF36). Of the 10 participants, HFLI TMS elicited a decrease in depressive symptoms in 7 participants, whereas 2 participants showed a slight increase in depression scores (2 and 3 points, respectively) and one patient had markedly increased depression score (13 points) before intervention. Regarding anxiety symptoms, 6 patients showed a decrease in symptoms, 2 showed increased symptoms but remained within normal range, one increased from 21 to 27 points, and one showed no changes. For general health, 5 subjects showed improvement in general health while 5 subjects showed a slight decrease in this measurement. There was no statistically significant median decrease in anxiety scores (−1.5 points) (*p* > 0.50), depression scores (−3.5 points) (*p* > 0.34), and SF36 scores (2 points) (*p* > 0.99). Changes before and after HFLI TMS are shown in Figure 2.

To explore if changes in BDI and BAI scores would be related to the mechanism in which subjects would stop smoking, we performed Spearman's rank-order correlation between changes from baseline in BDI and changes from baseline to week 8 in exhaled CO concentrations and between changes from baseline in BAI and changes from baseline to week 8 exhaled CO. There was no correlation between changes in depression and changes in exhaled CO (*r*_*s*_ = −0.13, *p* > 0.70) nor between changes in anxiety and changes in exhaled CO (*r*_*s*_ = .67, *p* > 0.85).

### 3.2. Discussion

Here, we report the case of ten patients who reached remission in tobacco consumption, measured both subjectively and objectively, after 24 sessions of HFLI TMS over the left prefrontal dorsolateral cortex. To our knowledge, this is the first report in which HFLI TMS has been applied to treat tobacco addiction.

The current paradigm in transcranial magnetic stimulation is that a high intensity magnetic field is necessary in order to change the brain's activity; however, this paradigm has been challenged by reports in which magnetic fields several orders of magnitude lower than current rTMS have biological and antidepressant effects [11, 21]; effect that we hypothesize has been seen in this study.

Several studies have shown that pharmacological treatment of depressive pathophysiology, even without clinical MDD, has an impact on addiction symptoms, and this effect can also occur with neuromodulation devices. Nonetheless, the exact mechanism in which magnetic fields of subthreshold intensity (not able to produce an action potential in the neuron) are able to produce biological action is currently unknown; however, this kind of magnetic pulses at frequencies higher than 50 Hz have shown to increase BDNF concentration [22] and brain's plasticity [23], which are well-known pathogenic mechanisms of MDD [24].

Taking this into account, we hypothesized that high frequency and low intensity magnetic pulses, similar to those that have shown antidepressant effect and that could be related to the dopaminergic pathways, could be useful in treating other pathological conditions that share neurobiological substrate, such as addictions; the same approach has been effective for drugs originally intended as antidepressants and later repurposed as a smoking cessation aid (e.g., bupropion) [25]; however, more studies are needed over the use of HFLI TMS with the same frequencies in order to determine the exact mechanism of action. It is important to emphasize that smoker patients included in this study showed moderate-to-high dependence to smoking, both physical and psychological. They were subjects who smoked more cigarettes than the average reported for Mexican population in national surveys (7 cigarettes per day) [26] and have tried to stop smoking several times. From the cessation point of view, they were smokers who could only achieve success by quitting, only with medication summed up to a behavioral intervention.

Regarding the results, HFLI TMS seems to start its action rapidly with most of the patients showing a remarkable reduction in CO levels just after one week of starting the intervention; however, statistical significance compared to baseline was first achieved after 3 weeks and later at 5, 6, and 7 weeks. The lack of statistical significance in the other time points could be explained by just one subject (subject 7); this patient showed a different behavior from the rest of the reported cases being the only one that struggled to diminish exhaled CO during the 8 weeks of stimulation and was one of the two patients who relapsed three months after treatment was over. Incidentally, the same subject had increased BDI and BAI scores by 13 and 6 points, respectively, while most of the subjects improved or remained in these clinimetric scores; even the other subject who relapsed three months after treatment was over showed an improvement in BDI and BAI scores at the end of treatment.

Most of the subjects showed improvement in BDI and BAI scores, which may indicate that, at least in those subjects, HFLI TMS could have helped subjects by reducing MDD symptoms; however, subjects that did not show any change or even increased BAI and BDI scores also lead to smoking cessation without significant changes in BAI and BDI scores; this could mean that HFLI TMS attacks other pathogenic mechanisms of addictions not necessarily related to depression. However, more studies, including neuroimaging and biomarkers, are required to elucidate the exact mechanism of action in HFLI TMS, especially in those subjects in which there is no change in depression and anxiety scores.

Besides its effectiveness in depression treatment, the application of HFLI TMS could represent a viable therapeutic option for nicotine addiction, as its low cost and the portability of the devices could ensure a better adherence to treatment, as it could be done in other settings as well as having a lesser impact in the patients' economy, resulting in better long-term outcomes as a result of better adherence. Also, the side effects reported by classic TMS devices can be severe (e.g., seizures), while HFLI TMS and related neuromodulation technologies have not reported these kinds of severe adverse effects.

However, it is essential to point out that the generalizability of our results could be affected as our sample size was small, as it was selected by convenience. Hence, no sample size calculations, nor observed power measures, were done, affecting the generalizability of our results. Also, because of the criteria used to select our sample, no participants that could not finish the required number of sessions were selected, which could exclude participants that experienced side effects and decided to suspend the treatment or participants that did not respond to treatment and abandoned it.

## 4. Conclusions

The present study shows that HFLI TMS could be an effective intervention for patients with moderate and heavy tobacco use who are trying to stop smoking even with previous history of failures in smoking cessation. However, our results should be regarded with caution, as this population is a small sample; even though inclusion and exclusion criteria were used, their individual differences could have affected the results of this study. Also, the study is retrospective and cases that fulfilled all criteria were selected after the treatment was finished, meaning that results could be biased as patients that had unsuccessful outcomes may have left the treatment before its conclusion. Additionally, it is important to note that due to the type of study conducted, there was no sham intervention applied so that placebo effect cannot be ruled out. While the observed effect was robust (ten out of ten subjects stopped smoking after the 8-week intervention and eight out of ten subjects remained this way 3 months after intervention), the external validity of our study is rather small, so conclusions in other populations cannot be drawn; a randomized double-blind clinical trial with an adequate sample size is required both to assess clinical efficacy and to elucidate possible mechanisms of action of HFLI TMS in the treatment of tobacco use disorder. Nonetheless, new tools to stop smoking are always welcome since smoking has proven to be one of the most difficult addictions to overcome for both physicians and smokers.

## Figures and Tables

**Table 1:** Clinical and epidemiological data of reported subjects.

		Subject 1	Subject 2	Subject 3	Subject 4	Subject 5	Subject 6	Subject 7	Subject 8	Subject 9	Subject 10	Mean (SD)
	Gender	M	M	F	M	F	F	F	F	M	M	
	Age (years)	57.00	64.00	65.00	39.00	59.00	55.00	50.00	61.00	46.00	72.00	56.80 (9.75)
	Years smoked	27.00	46.00	48.00	21.00	39.00	37.00	20.00	43.00	31.00	57.00	36.90 (12.12)
	Cigarettes/day	30.00	10.00	15.00	20.00	8.00	20.00	40.00	10.00	20.00	18.00	19.10 (9.80)
	Packets/year	40.50	23.00	36.00	21.00	15.60	37.00	40.00	21.50	31.00	51.30	31.69 (11.21)
	Attempts to quit smoking	1.00	0	3.00	2.00	3.00	2.00	1.00	2.00	1.00	5.00	2.00 (1.41)
	BMI	30.01	23.18	36.07	26.89	25.78	23.13	23.08	25.02	29.13	25.14	26.74 (4.07)
	FTND score	9.00	2.00	7.00	3.00	1.00	7.00	9.00	3.00	7.00	8.00	5.60 (3.03)
	TAPD score	59.00	32.00	53.00	39.00	39.00	48.00	59.00	52.00	63.00	58.00	50.20 (10.42)
	AUDIT score	4.00	0	2.00	1.00	1.00	1.00	2.00	0	5.00	0	1.60 (1.71)
BDI	Baseline	20.00	1.00	15.00	4.00	10.00	27.00	19.00	12.00	17.00	4.00	12.9 (8.28)
	Post-HFLI TMS	22.00	0	9.00	0	7.00	18.00	32.00	2.00	1.00	7.00	9.80 (10.83)
BAI	Baseline	9.00	0	15.00	0	1.00	23.00	21.00	18.00	2.00	25.00	11.40 (10.17)
	Post-HFLI TMS	8.00	1.00	7.00	0	6.00	16.00	27.00	0	0	2.00	6.70 (8.76)
SF36%	Baseline	64.75	88.52	90.16	98.36	95.08	82.79	90.16	92.62	93.44	68.03	86.39 (11.35)
	Post-HFLI TMS	84.43	86.07	98.36	93.44	90.16	98.36	95.08	87.70	91.80	88.52	91.39 (4.89)
CO ppm	Baseline	30	5	22	34	9	50	19	2	10	22	20.3 (14.81)
	Post-HFLI TMS	0	0	0	1	0	0	8	0	3	1	1.3 (2.54)

BMI, body mass index; FTDN, Fagerström Test for Nicotine Dependence; TAPDS, Test to Assess the Psychological Dependence on Smoking; AUDIT, Alcohol Use Disorders Identification Test; BDI, Beck Depression Index; BAI, Beck Anxiety Index; SF36%, percentage of Short Form Health Survey; CO ppm, parts per million of carbon monoxide; HFLI TMS, High Frequency and Low Intensity Transcranial Magnetic Stimulation.

## Data Availability

Anonymized data of the applied scores are available upon request.
